# Patient-specific hemodynamic assessment using experimental and computational approaches in anomalous aortic origin of the coronary artery: Case study

**DOI:** 10.1016/j.xjtc.2026.102378

**Published:** 2026-04-03

**Authors:** Thangam Natarajan, Yasaman Farsiani, Jayanthi Parthasarathy, Silvana Molossi, Carlos M. Mery, Michael Jiang, Joanna Ghobrial, Athar M. Qureshi, Tara Karamlou, Lakshmiprasad Dasi, Rajesh Krishnamurthy

**Affiliations:** a Wallace H Coulter Department of Biomedical Engineering, Georgia Institute of Technology, Atlanta, Ga; b Department of Radiology, Nationwide Children's Hospital, Columbus, Ohio; c Division of Cardiology, Department of Pediatrics, Texas Children's Hospital, Baylor College of Medicine, Houston, Tex; d Division of Pediatric Cardiac Surgery, Pediatric Heart Institute, Vanderbilt University Medical Center, Nashville, Tenn; e Division of Pediatric and Congenital Cardiac Surgery, Department of Thoracic and Cardiovascular Surgery, Cleveland Clinic, Cleveland, Ohio; f Department of Cardiovascular Medicine, Cleveland Clinic, Cleveland, Ohio; g Department of Pediatric Cardiac Surgery, Akron Children's and Cincinnati Children's Hospital, Akron, Ohio

**Keywords:** AAOCA, CFD, experiments, 3D-printing, hemodynamics

## Abstract

**Objective:**

To develop and demonstrate the feasibility of a patient-specific framework combining experimental and computational modeling for noninvasive hemodynamic evaluation of anomalous aortic origin of coronary artery (AAOCA) anatomy derived from coronary computed tomography angiography.

**Methods:**

A patient with a left AAOCA who experienced aborted sudden cardiac death underwent an unroofing procedure and later required coronary reimplantation after recurrent aborted sudden cardiac death was studied. Patient-specific models were generated at 3 stages: preoperative, postunroofing, and postreimplantation. Experimental assessment used 3-dimensional–printed aortocoronary models integrated with a pulsatile flow loop, whereas computational simulations were performed using computational fluid dynamics. Fractional flow reserve (FFR) and flow velocities were evaluated at rest and under simulated stress and compared with clinical stress FFR obtained during cardiac catheterization.

**Results:**

Clinical cardiac catheterization-derived FFR under dobutamine stress was 0.77 postunroofing and 0.99 after reimplantation. Experimental testing demonstrated similar stress FFR of 0.7 in native left AAOCA (with short intramural course), 0.8 after unroofing (compression from the intercoronary pillar), and 0.9 postreimplantation. Computational FFR agreed with both experimental and clinical measurements: lowest values were 0.74 preoperatively, 0.8 post unroofing, and 0.87 post reimplantation. Flow patterns and pressure gradients corresponded with surgical and anatomical observations, showing improved coronary perfusion after reimplantation.

**Conclusions:**

This hybrid experimental-computational workflow enables patient-specific assessment of AAOCA hemodynamics across sequential surgical stages. The approach provides accurate evaluation of coronary anatomy, cross-validation of model results, and preliminary mechanistic insight into ischemia, supporting the feasibility of this hybrid approach for patient-specific hemodynamic evaluation and motivating future investigation in larger cohorts.

## Graphical Abstract

**Figure:**
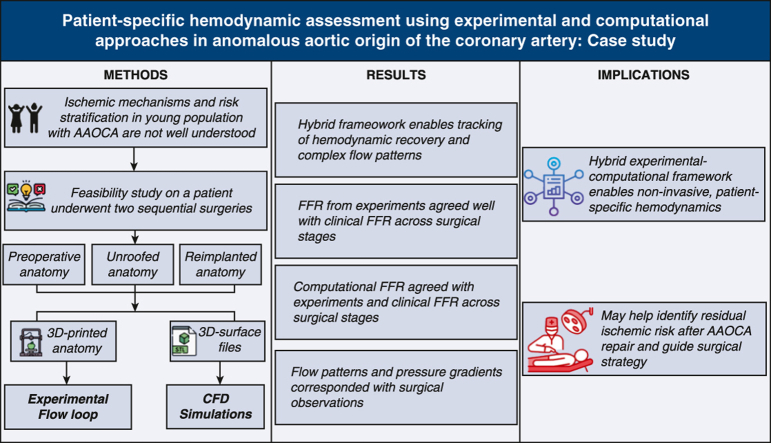

**Figure:**
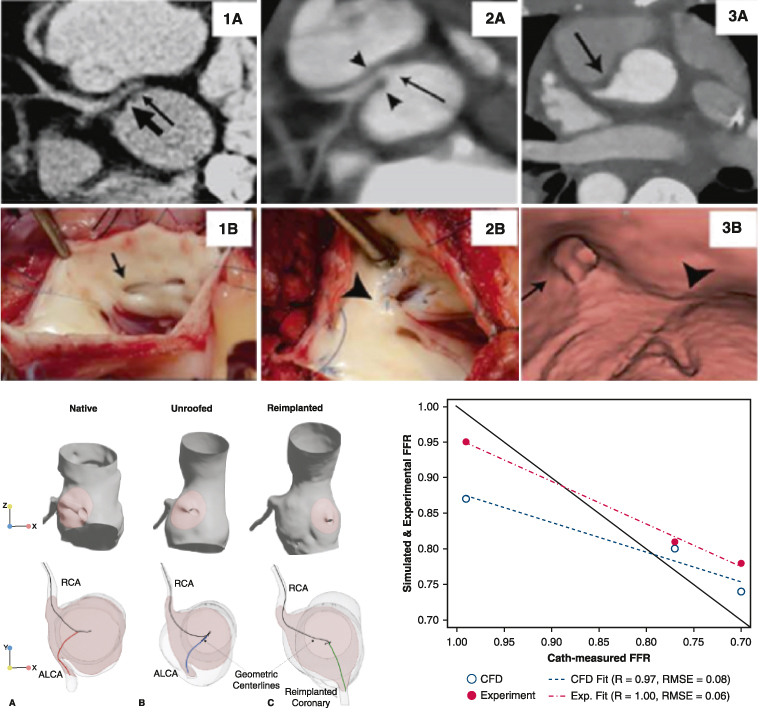
Hybrid 3D-printed flow loop & CFD framework assessing AAOCA hemodynamics across surgeries.

**Central Message**

A patient-specific hybrid framework of 3D-printed flow loops and CFD noninvasively quantifies coronary hemodynamics in AAOCA, enabling functional assessment and surgical planning.

**Perspective**

Patient-specific evaluation of anomalous coronary arteries remains challenging using anatomy alone. This study demonstrates how integrating experimental flow loops with computational modeling can noninvasively characterize coronary hemodynamics across surgical interventions, providing functional insight that may complement imaging and inform management in selected patients with AAOCA.

Young individuals with anomalous aortic origin of a coronary artery (AAOCA)—a rare congenital cardiac anomaly—face a heightened risk of sudden cardiac death (SCD), especially when the anomalous coronary arises from the opposite aortic sinus with an interarterial course (between the 2 great arteries), either with a single ostium or 2 separate ostia.^1^^,^^2^ The exact mechanism leading to SCD remains unclear but is thought to involve proximal coronary narrowing or hypoplasia due to an abnormal intramural course within the aortic wall (intramural course), which limits coronary reserve during exercise through dynamic compression. Affecting an estimated 0.03 to 0.9% of the population, AAOCA is the second-leading cause of SCD in young individuals.^3^ Among its variants, left anomalous aortic origin of a coronary artery (L-AAOCA) carries a greater risk of myocardial ischemia and adverse cardiac events than the more common anomalous right AAOCA.^4^^,^^5^ However, not every individual with AAOCA experiences SCD, making risk stratification a major clinical challenge, because management decisions depend on it.

The mere presence of AAOCA does not warrant exercise restriction or surgery; a comprehensive anatomic and hemodynamic evaluation is essential before intervention.^6^ The anatomical complexity of AAOCA, combined with patient-specific physiology, poses challenges in assessing SCD risk and guiding clinical management. These variations can markedly alter coronary blood flow, particularly during exercise, when the interplay between abnormal anatomy and myocardial demand may provoke ischemia without obvious precipitating factors.^7^ Clinical decision-making is further complicated by the absence of standardized management strategies, the limited yield of conventional stress imaging, and the infrequent use of invasive functional testing in children.

Current guidelines often rely on anatomical markers for surgical decisions, but these do not account for the flow mechanisms underlying ischemia and SCD.^8^ Residual high-risk anatomical features after AAOCA repair are not uncommon,^9^ yet their physiological significance remains uncertain, complicating return-to-activity decisions. Several hypotheses have linked coronary anomalies to ischemia on the basis of features such as slit-like ostia, abnormal take-off angles, high ostial location, proximal hypoplasia, and intramural course length.^10^, ^11^, ^12^, ^13^ However, few studies have rigorously explored how these anatomical factors contribute to SCD. Therefore, there is a pressing need for advanced in vitro and in silico techniques that can replicate the complex hemodynamics of AAOCA in both pre- and postoperative states, enabling patient-specific risk assessment and more informed management strategies.

Recent advances in 3-dimensional (3D) printing, in vitro modeling, and computational fluid dynamics (CFD) have expanded the ability to study patient-specific hemodynamics in coronary anomalies. Previous experimental work has shown the feasibility of constructing physiologic flow loops with computed tomography angiography (CTA)-derived 3D-printed aortocoronary models, allowing direct measurement of flow and pressure under controlled conditions.^14^^,^^15^ In parallel, CFD studies have been used to probe coronary flow and wall mechanics with high spatial and temporal resolution.^16^, ^17^, ^18^, ^19^ Each modality offers unique advantages: physical models provide experimental validation and compliance effects, whereas simulations enable detailed mechanistic analysis.

In this work, we present a unified framework integrating both flow loop experiments with 3D-printed anatomy and patient-specific CFD simulations to evaluate hemodynamics across preoperative and 2 sequential surgical stages (native, unroofed, and reimplanted) in the same patient with AAOCA. This feasibility case study demonstrates how combining in vitro and in silico methods can enhance understanding of coronary flow dynamics across sequential surgical anatomies in patients with AAOCA.

## Methods

### Patient Case Overview

The investigation is a retrospective analysis of a patient who underwent interventions for L-AAOCA. The institutional review board approved the study (IRB ID: STUDY00000525, initial date of approval: July 29, 2020), and the need for informed consent was waived. The clinical details and surgical history of this patient, including the unroofing and subsequent reimplantation procedures, have been previously reported.^20^ Key details are summarized to follow for completeness.

**Figure 1:**
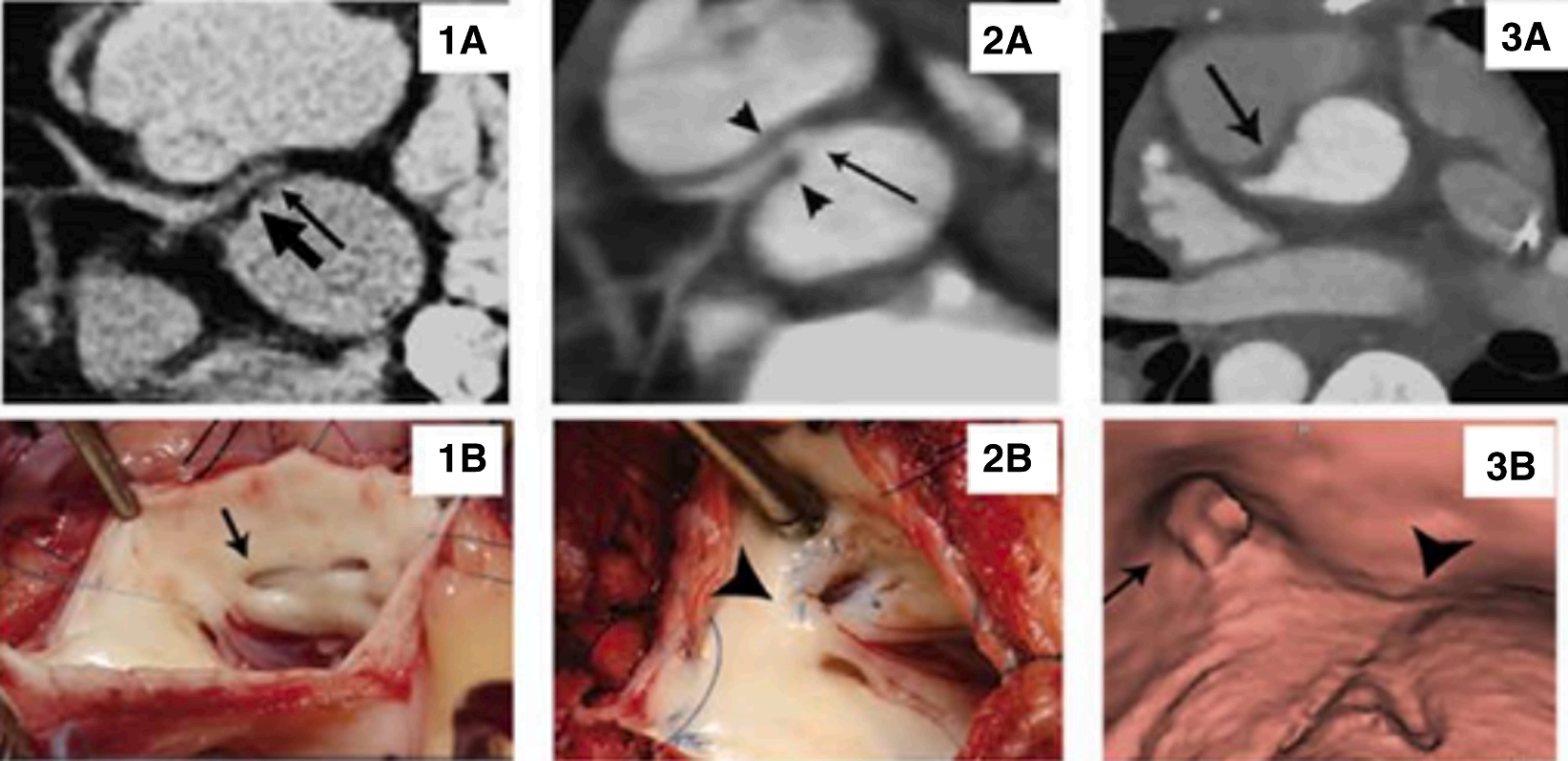
Imaging and surgical findings from a patient with a left anomalous aortic origin of a coronary artery from the right sinus of Valsalva and a short 3.5-mm intramural course before initial repair (column 1), postunroofing (column 2), and postcoronary transection and reimplantation (column 3). Initial preoperative (A) and intraoperative (B) views show a slit-like ostium (*thin arrow*). After initial unroofing, imaging (A) and intraoperative (B) views show an increase in the size of the ostium (*thin arrow*) but persistent compression by the intercoronary pillar (*arrowheads*) remains. After coronary reimplantation, axial imaging (A) and angioscopic view (B) show a wide-open ostium (*arrow*) away from the intercoronary pillar (*arrowhead*).

Reproduced with permission from Agrawal and colleagues.^20^

A 12-year-old male athlete experienced sudden cardiac arrest while playing basketball and was successfully defibrillated. Coronary CTA revealed an L-AAOCA arising from the right sinus near the intercoronary commissure, with a stenotic orifice and a short 3.5-mm intramural segment. During surgery, the intramural segment was unroofed up to the intercoronary pillar, leaving the left coronary artery (LCA) still arising from the right sinus (Figure 1). Follow-up dobutamine stress cardiac magnetic resonance imaging performed 3 months later was normal, with no inducible perfusion abnormalities, and he returned to full activity. Twenty months postoperatively, he experienced recurrent cardiac arrest, was successfully resuscitated, and received an implantable cardiac defibrillator. Repeat CTA showed a wide open LCA neo-ostium from the right sinus, but proximal narrowing due to the intercoronary pillar, and a previously unrecognized 1.9-cm myocardial bridge of the left anterior descending (LAD) artery. Cardiac catheterization with intravascular ultrasound assessment showed 65% narrowing in the proximal LCA, with fractional flow reserve (FFR) declining from 0.9 at rest to 0.77 under dobutamine stress and 0.65 in the intramyocardial LAD segment, indicating compromised coronary flow at both sites. Reoperative findings confirmed that the proximal LCA coursed behind a prominent intercoronary pillar, causing stenosis. The LCA was transected and reimplanted into the left sinus of Valsalva, and the LAD myocardial bridge was unroofed. A postoperative CTA at 3 months showed a normal LCA origin with no stenosis, findings confirmed by repeat FFR and intravascular ultrasound. Since returning to full activity, the patient's clinical course has been uneventful.

### Image Acquisition and 3D-Model Construction

**Figure 2:**
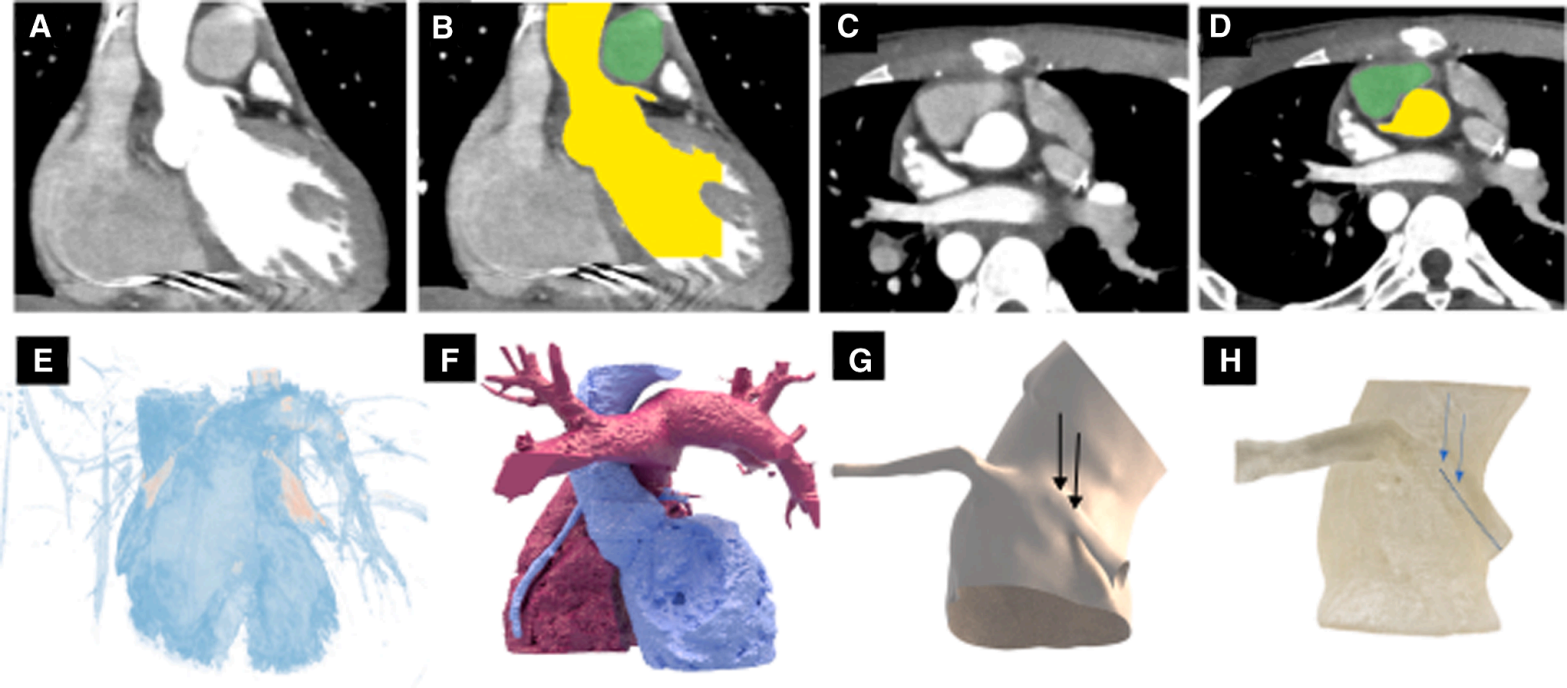
Patient-specific 3-dimensional (*3D*)-model construction pipeline for anatomical and functional assessment of coronary anomaly. A-D, Computed tomography angiography (*CTA*) images from different planes showing (A and C) raw grayscale slices and (B and D) segmented anatomical features, including the aorta (*yellow*) and pulmonary artery (*green*). E, Volume-rendered whole-heart visualization for anatomical context. F, 3D reconstruction of ventricles and vasculature from segmented CTA data, showing the aorta (*red*), pulmonary artery (*blue*), and coronary arteries. G, The surface-rendered model highlights anomalous coronary origin and proximal course (*black arrows*). H, 3D-printed anatomical model with visualized coronary course (*blue arrows*) used for bench-top or flow experiments.

The patient's data were evaluated at 3 stages: preoperative (native anatomy), postunroofing, and postreimplantation. At all 3 stages, electrocardiogram-gated CTA was performed using a 320-row volumetric scanner (Aquilion ONE, Canon Medical Systems). Retrospective electrocardiogram gating and half-scan reconstruction generated 20 phases at 5% intervals across the cardiac cycle. Manual review of all phases was performed, and, if needed, additional incremental phases were reconstructed to identify the best phase with minimal cardiac and coronary motion for image reconstruction.^21^ CTA-DICOM datasets were segmented using a previously described algorithm to produce 3D surface models in stereolithographic (STL) format. STL files were edited in MeshLab, an open-source 3D computer-aided design program, to smooth surfaces and remove artifacts, serving as inputs for both the 3D printing and computational modeling (Figure 2). Print material composition and wall thickness of the aorta and coronaries were assigned on the basis of previous testing to match the mechanical properties and compliance of fresh pediatric human aorta, whereas the intimal wall thickness of the intramural segment was derived directly from the CTA. The 3D-printed aortocoronary models were mounted in an aortic position within a box and embedded in Gelly Wax (RBC Industries) to simulate the mediastinal environment. STL files were imported into Objet Studio and printed on a Connex 3 Objet printer using Agilus30 material. Support material was removed by immersion in a sodium hydroxide bath, and models were coated externally with a thin layer of polyurethane to improve visual clarity and durability. For additional details on material testing and selection, readers are referred to Parthasarathy and colleagues.^15^

### In Vitro Circulatory Flow Loop

**Figure E1:**
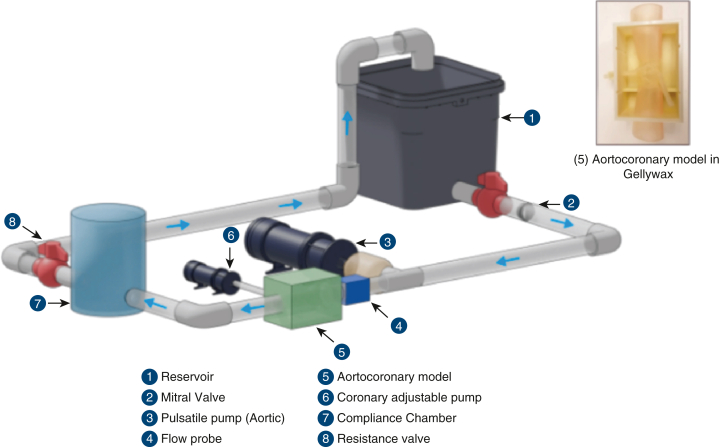
Schematic of the pulsatile flow loop replicating left-heart and coronary physiology. The loop includes a reservoir (1), mitral valve (2), pulsatile aortic pump (3), flow probe (4), aortocoronary model in Gellywax (5), coronary-specific adjustable pump (6), compliance chamber (7), and adjustable resistance valve (8). Flow direction is indicated with blue arrows. An inset shows the embedded aortocoronary model in Gellywax.

A validated pulsatile left-heart simulator was used to test the 3D-printed biomechanical models (schematic in Figure E1). The bladder pump, controlled by a LabVIEW program, drove the main flow throughout the cardiac cycle. Aortic and coronary flows were measured with ultrasonic flow probes (ME 25 PXN, Transonic Systems Inc). Distal coronary resistance for the LCA was incorporated using a Sterling-type controller. The flow loop included a mechanical prosthetic valve (surrogate for the mitral valve) between the reservoir and bladder pump, the aortocoronary model, and a compliance chamber with adjustable pressure to reproduce systemic compliance. Dynamic pressures within the models were recorded at 100 Hz using a Millar Mikro-Tip catheter (0.84 Fr; Millar Instruments) and ensemble-averaged over 100 cardiac cycles. The working fluid was a 3.5 cSt water–glycerin mixture (≈36% glycerin; density = 1060 kg/m^3^), matching the kinematic viscosity of blood. The flow loop was tuned to the patient's cardiac output by adjusting resistances and compliances in both coronary and systemic circuits. A stress condition was simulated by elevating aortic pressure to ≈200 mm Hg to evaluate pressure-induced anatomical deformation and its effect on coronary flow. The pressure–flow slope was quantified by maintaining constant cardiac output while progressively increasing aortic pressure and continuously recording coronary flow rate.

### CFD Simulations

**Figure E2:**
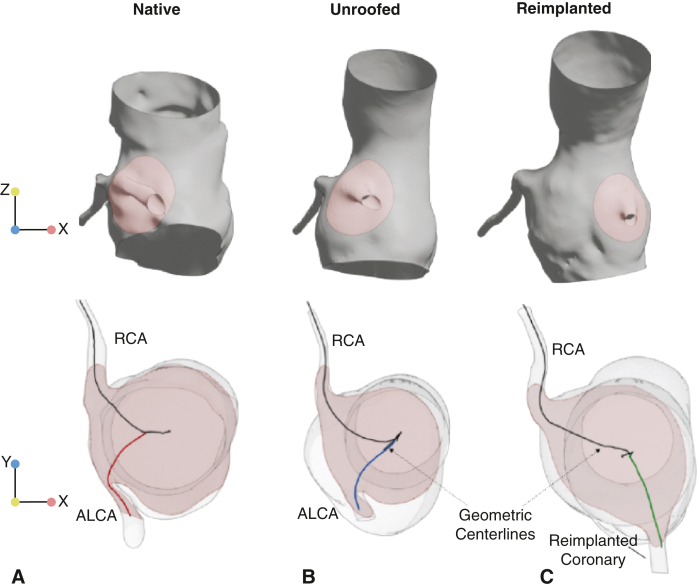
Surface models show (A) native, (B) unroofed, and (C) reimplanted anatomies and the lower panel showing their respective geometric centerlines.

The segmented models for the preoperative and 2 postoperative anatomies are shown in Figure E2. The lower panel overlays geometric centerlines to illustrate vessel curvature and orientation relative to the sinus and sinotubular junction. Blood in large vessels was modeled as a Newtonian fluid, because non-Newtonian effects are negligible under high rates. Large eddy simulations were performed using the open-source finite-volume software OpenFOAM 12.0 (OpenFOAM Foundation). Each geometry was discretized into ≈4 million hexahedral cells using blockMesh and snappyHexMesh. Mesh convergence was confirmed by doubling resolution to ≈8 million cells, yielding <2% variation in FFR. Local refinement was applied near coronary ostia to resolve flow in the narrow intramural segments. Time discretization used a second-order backward implicit scheme, and convection terms used a second-order central differencing scheme. The time step ensured a Courant-Friedrichs-Lewy number <1.5. Pressure–velocity coupling was handled with the PIMPLE algorithm. Simulations were executed on the PACE Phoenix cluster (dual Intel Xeon 6226 @ 2.7 GHz CPUs), each spanning 5 cardiac cycles and requiring ≈6 hours of computation using 28 cores. Rigid, no-slip walls were assumed, with fluid density equal to 1060 kg/m^3^ and kinematic viscosity equal to 3.5 cSt. The inlet aortic waveform was derived from the in vitro experiments (see the section “In Vitro Circulatory Flow Loop”), and 3-element Windkessel type resistances were applied at the outlets, with resistances and compliances based on loop measurements and implemented via custom boundary conditions following Manchester and colleagues.^22^ In this analysis, FFR is reported as the instantaneous pressure ratio (P_d_/P_a_) across the cardiac cycle rather than the cycle-averaged clinical metric, allowing visualization of temporal pressure variations consistent with the computational framework.

## Results

### In Vitro Results

**Figure 3:**
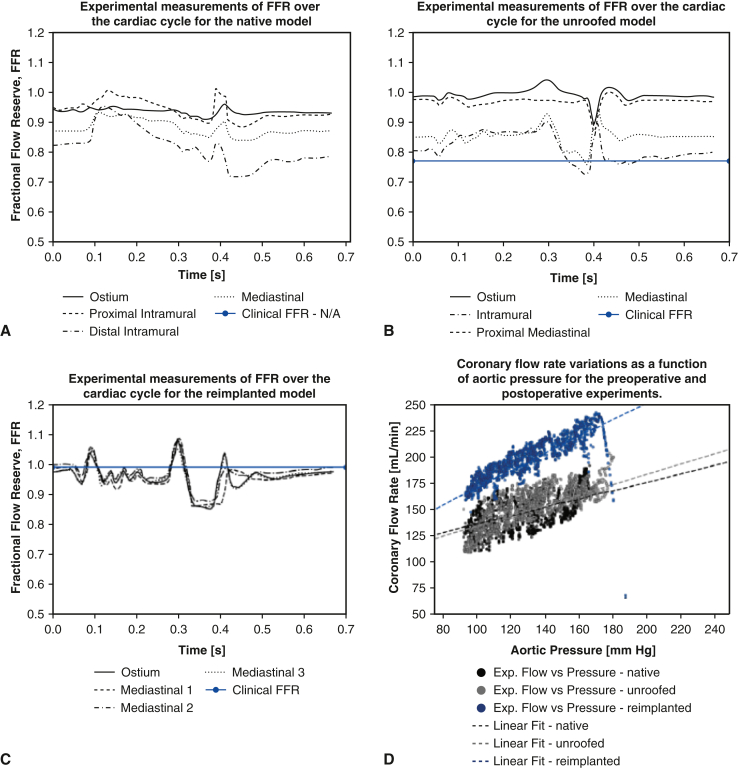
Experimental measurements of resting fractional flow reserve (*FFR*; P_d_/P_a_) variations versus time for (A) native (preoperative), (B) unroofed, and (C) postreimplantation models at different locations along the proximal left coronary artery and in (D) measured coronary flow variations from experiments as a function of pressure for the 3 models.

Time-resolved FFR data across the cardiac cycle for the preoperative and 2 postoperative stages are shown in Figure 3. The minimum FFR decreased to 0.78 in the distal intramural segment preoperatively, consistent with ostial stenosis (Figure 3, *A*) and remained reduced at 0.81 after unroofing just distal to the intercoronary pillar (Figure 3, *B*), indicating persistent risk for dynamic narrowing and incomplete relief of ischemia. After reimplantation into the correct sinus, FFR values normalized (>0.9) across all sites (Figure 3, *C*), suggesting restoration of unobstructed coronary flow. The timing and location of FFR drops throughout the cardiac cycle were consistent with intraoperative surgical findings.

Under simulated stress testing, the relationship between coronary flow rate and aortic pressure exhibited distinct patterns across surgical stages (Figure 3, *D*). Linear fits applied to the pressure–flow data revealed progressively steeper slopes with each intervention, ie, 0.25 preoperatively, 0.31 postunroofing, and 0.50 postreimplantation, indicating a sequential reduction in coronary resistance. The increased slope after reimplantation confirms improved perfusion capacity and a more physiologic pressure–flow response.

### CFD Results

**Figure 4:**
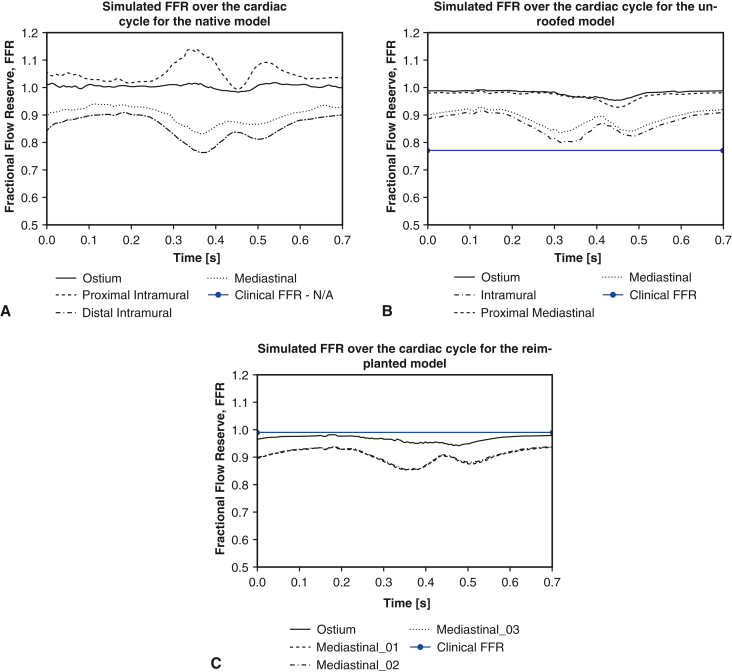
Simulated resting fractional flow reserve (*FFR*) variations versus time for (A) native (preoperative), (B) unroofed, and (C) postreimplantation models at different locations along the proximal left coronary artery. In the native model, the lowest FFR obtained was 0.74. After unroofing, the lowest FFR was 0.8, and after reimplantation, the FFR was 0.87.

**Figure 5:**
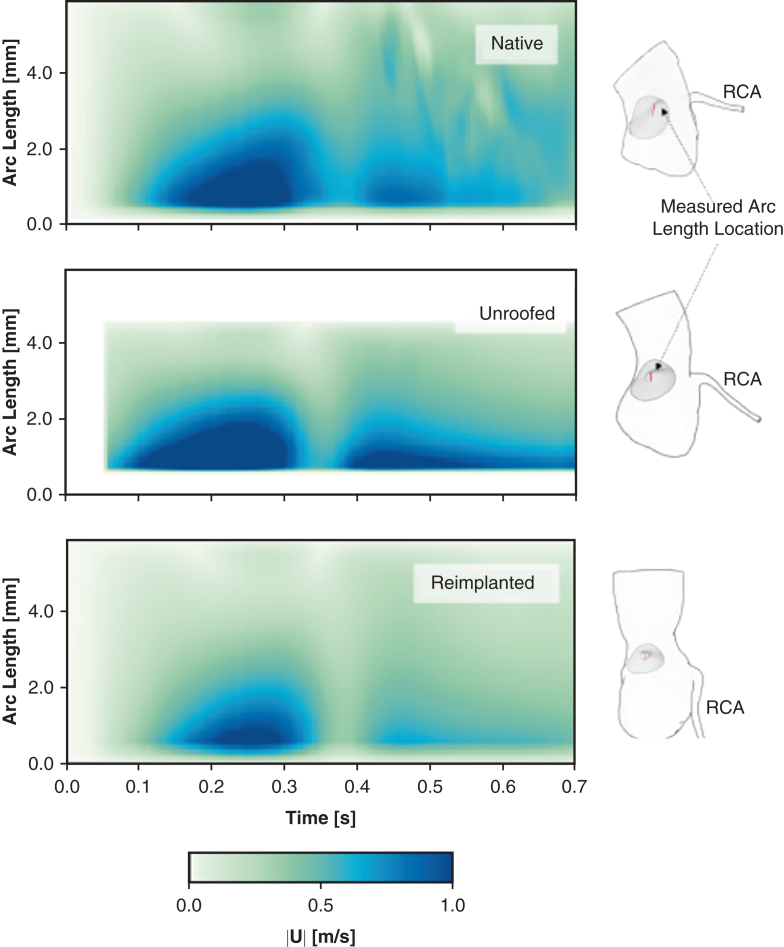
Spatiotemporal velocity maps along a defined arc at the left coronary artery ostium across surgical stages: native, unroofed, and reimplanted. The *vertical axis* represents the centerline position from the ostial segment, and the *horizontal axis* represents time over one cardiac cycle. *Color* indicates instantaneous velocity magnitude in m/s. In the native case, velocity begins to increase during systole and reaches maximum throughout the systolic phase due to the reduced area or constriction of the intramural segment. The magnitude of velocity decreases moderately after the unroofing procedure but remains relatively high throughout systole, particularly in the lower half of the arc length. The reimplanted space-time plots show that the velocity is much lower and within the normal physiological range of coronary flow.

**Figure E3:**
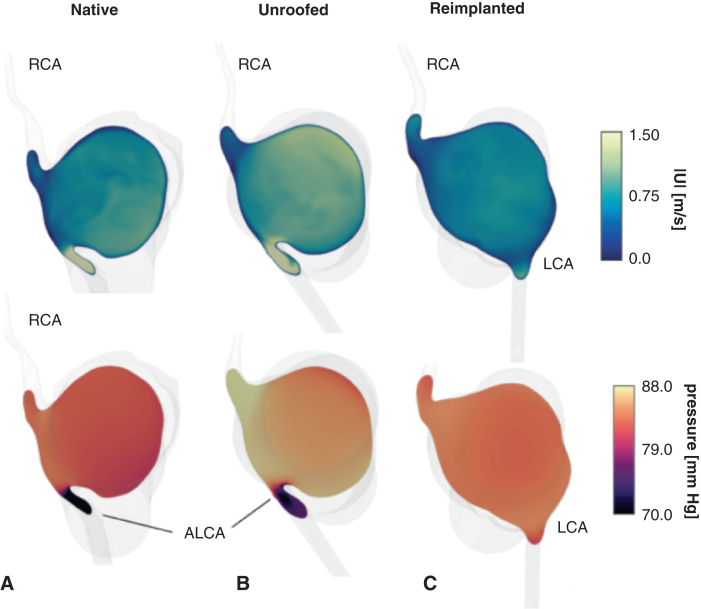
Velocity magnitude and pressure of (A) Native, (B) unroofed, and (C) reimplanted anatomies on an axial slice along the centerline of their respective geometries.

**Figure E4:**
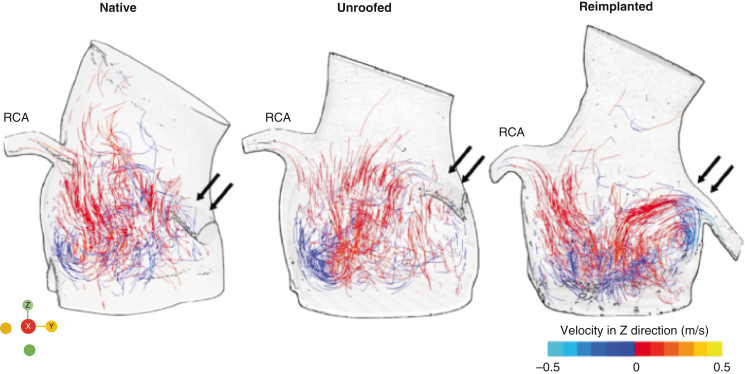
Snapshot of pathlines experiencing constriction in the first two cases as opposed to the reimplanted case (see Supplemental Video 1).

Computational FFRs for the native and 2 postoperative stages are shown in Figure 4, *A-C*. In the preoperative model, the lowest FFR within the cardiac cycle was 0.74, which increased to 0.80 postunroofing and 0.87 after reimplantation. Figure 5 illustrates space-time velocity plots across the ostium for all 3 cases, where velocity magnitude is mapped along a fixed line throughout the cardiac cycle. Figures E3 and E4 present corresponding velocity–pressure fields and streamline visualizations, respectively. In the first 2 anatomies, regions of elevated velocities and constricted streamlines coincide with anatomically narrowed segments that are associated with reduced FFR, ie, at the intramural segment in the preoperative case and the obstructing intercoronary pillar in the unroofed case. After reimplantation, velocity and pressure fields normalize, indicating restored flow (see Supplemental Video 1 for the visualization of streamlines throughout the cardiac cycle). Temporally, the FFR drop occurs near end-systole and rises during diastole, a trend consistent across all stages but differing in magnitude as reference pressures decrease downstream. The lowest (P_d_/P_a_) values align with the most stenotic regions: the distal intramural segment in the preoperative anatomy and the proximal mediastinal segment post-unroofing. Pressure recovery downstream corresponds to lower velocities in regions of increased cross-sectional areas.

### Comparison Between Clinical, Experimental, and Simulated FFR

**Figure 6:**
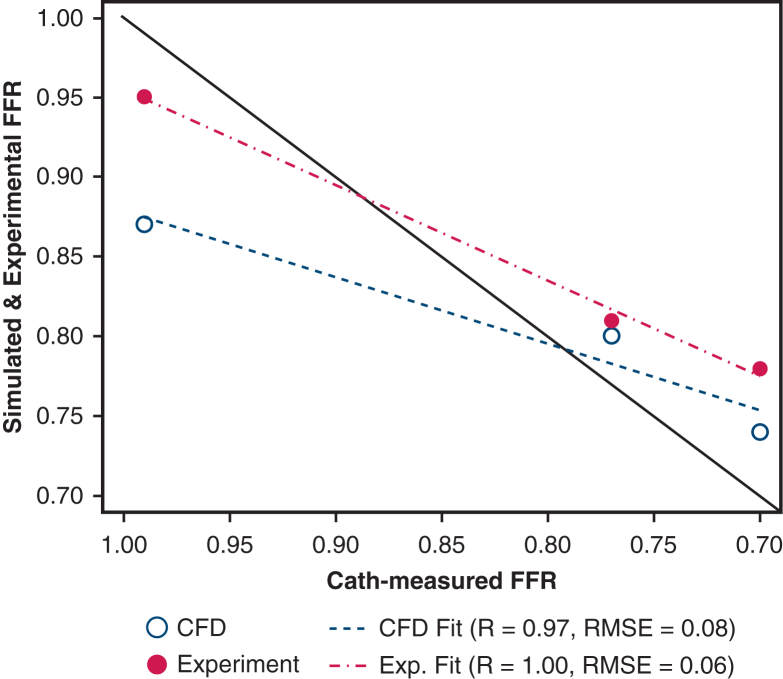
Correlations between experiments, simulations, and measured catheter-measured fractional flow reserve (*FFR*). *CFD*, Computational fluid dynamics.

Figure 6 shows the agreement between all 3 approaches to measuring FFR. The native anatomy exhibited significantly reduced FFR and elevated pressure gradients, suggesting compromised coronary perfusion. Both the experiments and the CFD confirmed this, with the experimental FFR dropping to 0.79 and the computational FFR also dropping to 0.79. After unroofing, partial improvement was observed, though flow recovery was limited by residual compression from the intercoronary pillar. The moderate improvements are reflected in FFR and lower velocities in the space-time flow plots. In the pathline flow visualizations, more particles exited through the unroofed coronary artery than in the native state, though the difference was modest. A clear improvement was seen only after reimplantation, when both models approached physiological FFR values and pressure gradients consistent with clinical measurements. These results demonstrate the hybrid model's ability to track hemodynamic recovery over time and offer insight into why unroofing alone may not always yield complete improvement.

## Discussion

This case study presents a patient-specific framework combining in vitro and in silico methods to evaluate hemodynamic changes across sequential surgeries in AAOCA. Using 3D printing, a pulsatile flow loop, and CFD modeling, we examined FFR, flow distribution, pressure contours, and pathlines across native, unroofed, and reimplanted anatomies in a patient who experienced aborted sudden cardiac arrest before and after initial surgery. The 2 approaches showed consistent trends, revealing pressure recovery and improved coronary flow after the first repair, but residual hemodynamic compromise that resolved following reimplantation. To our knowledge, this is among the first reports integrating physical and computational models to noninvasively examine hemodynamic changes across surgical stages in AAOCA.

Most noninvasive methods rely on estimated boundary conditions that may not reflect patient physiology. By tuning the experimental setup to match patient-specific cardiac output and coronary flow waveforms, resistance values were directly incorporated as CFD boundary conditions, enabling more physiologic simulations. Small differences between clinical, experimental, and CFD-derived FFR values likely reflect uncertainties in segmentation, boundary conditions, and rigid-wall assumptions rather than full fluid–structure interaction. Razavi and colleagues^23^ report that the sensitivity to distal coronary resistances is high and causes significant differences in FFR predictions. Despite these limitations, the combined experimental-computational framework provides a physiologically grounded approach for interpreting patient-specific coronary hemodynamics.

In the current feasibility study, the integration of in vitro and in silico methods offers a robust, complementary framework for understanding the complex hemodynamics of AAOCA across surgical stages. In vitro experiments offer validation for computational predictions, reproducing realistic flow patterns, in physical replicates of patient-specific anatomies, as demonstrated in recent case studies.^24^ In parallel, CFD enables detailed analysis of flow patterns, pressure fields, wall shear stress, and resistance distribution data that are difficult to capture invasively and experimentally. Together, they strengthen interpretation, offer cross validation, and build confidence in applying this methodology to clinical scenarios. First, experimental flow loop measurements serve as a critical validation tool for computational predictions, ensuring physiologic accuracy of simulated metrics such as FFR, pressure drops, and flow splits, which are critical in the preoperative and unroofed states where intramural compression and residual narrowing may confound interpretation. Second, CFD uniquely enables access to internal variables (with high spatial and temporal resolution) such as velocity fields and localized pressure gradients that are not directly measurable in the experimental flow loop but could be essential for understanding ischemic risk in repaired anatomies. For example, the space-time plots shown earlier indicate the presence of flow abnormalities, which are consistent with clinical observations reported by Farooqi and colleagues.^25^ Third, simulations enable systematic manipulation of boundary conditions such as changes in distal coronary resistance or exercise-induced stress, facilitating hypothesis testing that would be difficult or impractical to achieve with physical models. Fourth, the experimental setup contributes to improved physiologic relevance by reproducing dynamic coronary flow waveforms and compliance, which in turn improves the fidelity of the boundary conditions used in simulations. Finally, this hybrid approach facilitates a feedback loop for surgical planning, allowing for a comparative analysis of unroofing versus reimplantation strategies in the same patient and across stages.

Segmented 3D geometries derived from a single CTA dataset serve as the common starting point for both experimental fabrication and CFD simulations, enabling a noninvasive, reproducible workflow. In principle, such integrated modeling approaches could help explore mechanisms of residual obstruction after repair; however, broader validation across larger patient cohorts will be necessary before clinical application. Predicting physiologic consequences across anatomical configurations can also aid virtual surgical optimization and personalized planning. While the workflow currently requires ≈3 to 4 days, (including 3D printing, flow loop tuning, and CFD simulations) it remains clinically actionable. As the process becomes automated, faster implementation could enable integration into preoperative planning for selected AAOCA patients. Beyond immediate utility, the framework provides a foundation for future predictive models of ischemia in AAOCA and related pathologies.

Despite its strengths, the current approach has limitations. CFD simulations assumed Newtonian rheology, rigid walls, and constant viscosity, potentially underrepresenting physiologic complexity. The model did not account for microvascular resistance or myocardial perfusion heterogeneity, and the analysis was limited to a single case. In addition, because this report reflects a single patient with a complex postoperative course, the findings should be interpreted as a proof-of-concept demonstration rather than validation of the modeling framework. Future work should include parameter-tuning methods^26^ and multiscale models coupling coronary flow and pressure^27^ to improve physiologic fidelity. Although these simplifications may affect quantitative accuracy, the consistency between experimental and computational findings supports the robustness of this hybrid framework. Future directions include expanding the cohort, incorporating fluid–structure interaction for wall motion and compliance, and refining outflow boundary conditions to better capture coronary perfusion dynamics.

## Conclusions

We developed and applied a hybrid experimental–computational platform to assess coronary hemodynamics in L-AAOCA across preoperative, unroofed, and reimplanted anatomies in a single patient. The 3D-printed circulatory flow loops and CFD models showed good agreement with clinically measured FFR across the 2 operative stages (preoperative FFR was unavailable following the initial aborted SCD). Cross-validation between these independent engineering approaches strengthens confidence in the model's noninvasive prediction of FFR. The physiologically grounded, patient-specific models provided detailed hemodynamic insights, elucidating the potential mechanisms underlying ischemia. This case report demonstrates the feasibility of combining in vitro flow loops and CFD to characterize patient-specific coronary hemodynamics across sequential surgical anatomies in AAOCA. The concordance between experimental, computational, and clinical FFR measurements supports this hybrid framework as a proof-of-concept platform for future investigation in larger cohorts.

## Conflict of Interest Statement

The authors reported no conflicts of interest.

The *Journal* policy requires editors and reviewers to disclose conflicts of interest and to decline handling or reviewing manuscripts for which they may have a conflict of interest. The editors and reviewers of this article have no conflicts of interest.

## Supplementary Data

Video 1 Visualization of streamlines throughout the cardiac cycle. Video available at: https://www.jtcvs.org/article/S2666-2507(26)00185-9/fulltext.
